# Anatomical considerations for safe retractor placement in distal biceps repair: a cadaveric study of the posterior interosseous nerve

**DOI:** 10.1007/s00276-025-03728-3

**Published:** 2025-10-06

**Authors:** Feras Qawasmi, Mark Goss, Fadi Shweiki, Steven Grindle, Mustafa Yassin

**Affiliations:** 1 Rabin Medical Center, Orthopedic Department, Hasharon Hospital, Petah Tikva, Israel; 2 Medical College of Wisconsin, Milwaukee, USA; 3 Saint Joseph Hospital, Jerusalem, Israel

**Keywords:** Radial nerve anatomy, Posterior interosseous nerve (PIN), Safe zone, Radial tuberosity, Distal biceps tendon repair, Retractor placement safety

## Abstract

**Purpose:**

Distal biceps tendon repair is a common procedure with a risk of iatrogenic nerve injury, including the posterior interosseous nerve (PIN). This study aimed to define the anatomical relationship of the PIN to key elbow landmarks, particularly the radial tuberosity, and to identify safe zones for retractor placement during surgery.

**Methods:**

Twenty-nine fresh cadaveric upper extremities were dissected by a fellowship-trained shoulder and elbow surgeon. Measurements included the bifurcation point of the radial nerve into the PIN and superficial radial nerve. We measured the PIN’s distance from the radiocapitellar joint (RCJ) and its relation to the radial tuberosity in both pronation and supination. The safe positioning of retractors relative to the radial tuberosity was also evaluated.

**Results:**

The radial nerve bifurcated approximately 5 ± 3 mm proximal to the RCJ. The PIN entered the supinator (Arcade of Frohse) at 22 ± 1 mm distal to the RCJ. It was located a mean of 13 ± 2 mm from the radial border of the radial tuberosity in pronation and 19 ± 2 mm in supination. In all specimens, the PIN distal to the arcade was surrounded by muscle, with no direct contact with bone. Safe retractor placement was ulnar to the tuberosity proximally and radial distally in supination.

**Conclusion:**

Supination increases the separation between the PIN and the tuberosity by ~ 5 mm, supporting ulnar proximal and radial distal retractor placement (narrow right-angled/Langenbeck) to reduce iatrogenic injury.

**Level of evidence:**

IV (Anatomical cadaveric study).

## Introduction

Distal biceps tendon ruptures frequently occur, particularly in middle-aged individuals. Surgical repair is often necessary to restore function and strength. While current surgical techniques have been shown to produce favorable outcomes in terms of strength and range of motion, they are not without risks [9, 13]. Reported complications include neurological injuries, most notably to the lateral antebrachial cutaneous nerve [2, 6], and the posterior interosseous nerve (PIN) [2, 3, 13]. Additional complications such as heterotopic ossification, radioulnar synostosis, and biceps tendon re-rupture have also been documented [2, 6, 14].

Distal biceps tendon repair is commonly performed through either a single-incision (SI) anterior approach or a double-incision (DI) technique [6, 14]. SI repairs offer direct access to the tuberosity but carry a recognized risk to the posterior interosseous and lateral antebrachial cutaneous nerves [2, 6, 11], whereas DI approaches may reduce anterior nerve risk yet have been associated with heterotopic ossification and radioulnar synostosis [6, 11–13].Regardless of approach, safe retractor placement and forearm positioning are critical.

**Fig. 1:**
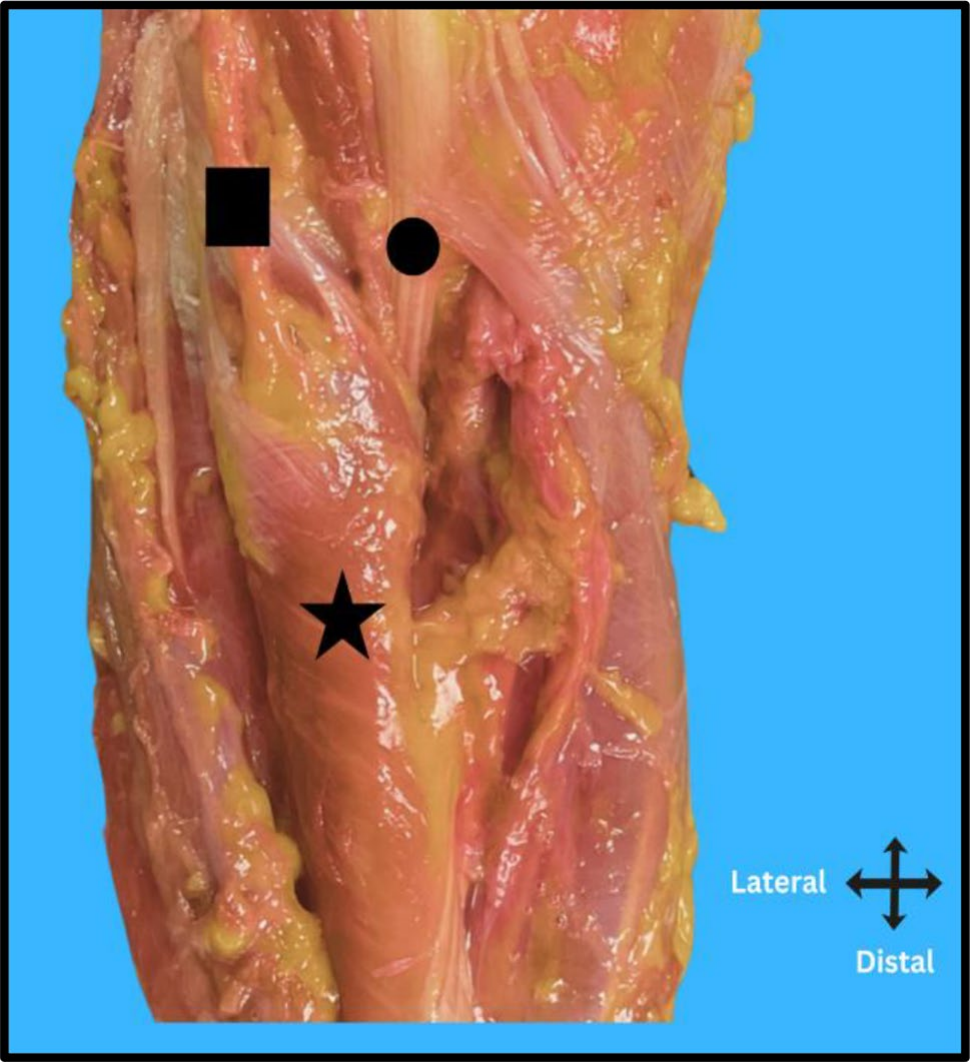
Cadaveric dissection showing the posterior interosseous nerve (PIN) marked with a square, the distal biceps tendon marked with a circle, and the supinator muscle marked with a star

The susceptibility of the PIN stems from its anatomical course (Fig. 1). The radial nerve bifurcates into the PIN and superficial radial nerve (SRN) near the lateral epicondyle, after which the PIN courses along the radius and enters the supinator muscle. The PIN provides motor innervation to the extensor muscles of the wrist and fingers, making it critical for hand [5], Iatrogenic injury can significantly impair a patient’s function and quality of life, potentially outweighing the benefits of the surgical procedure.

Recent work underscores considerations relevant to the radial nerve and particularly PIN, which possess rich vascularization, an average of 8 vascular afferents, which may increase susceptibility to traction or compression from retractors [8]. Variations in radial-nerve branching, as reported by Khadanovich et al., are a potential contributor to iatrogenic injury [16]; importantly, Abdullah et al. showed that high-resolution ultrasonography enables reliable preoperative identification of small peripheral nerves and their variants [1].

Numerous studies have examined the PIN’s location in relation to anatomical landmarks [4, 19, 23]. Most have focused on the bifurcation level of the radial nerve relative to the lateral epicondyle [4, 7, 21], the distance between the joint line and the supinator entry point of the PIN [21], or the radial head’s relation to the PIN as it crosses the radius[10, 15, 17]. Additionally, studies have analyzed how forearm pronation and supination affect these distances [10, 15]. These anatomical assessments are critical for defining safe zones during distal biceps tendon repair and proximal radius procedures [10, 15, 17].

Furthermore, two studies have specifically examined the distance between the distal biceps tendon and the Arcade of Frohse [22, 23]. while another MRI-based study evaluated the relationship between the PIN and the radial tuberosity, aiming to identify safe drill placement during distal biceps repair [19].

While prior anatomical and imaging studies have described the relationship between the posterior interosseous nerve (PIN) and the radial tuberosity, they provide limited operative guidance for retractor placement during distal biceps repair. We therefore used fresh cadaveric specimens to quantify the PIN’s relationship to surgical landmarks with the forearm in standardized positions and to derive retractor-specific safe zones.

## Materials and methods

Twenty-nine fresh cadaveric upper extremities were dissected (17 male, 12 female; age mean: 68.5 years, range: 59–78 years; 15 right, 14 left) by a single orthopedic shoulder and elbow surgeon. The dissections were performed using 2.5 × magnification loupes to enhance visualization. The cadaveric specimens were obtained through the Body Donation Program under donor-written consent. Ethical approval for anatomical studies was waived by the Institution in accordance with regulations for cadaveric research. They were thawed at room temperature for 12–18 h before dissection. Limbs with previous surgery, deformity, or gross pathology around the elbow/forearm were excluded after inspection.

**Fig. 2:**
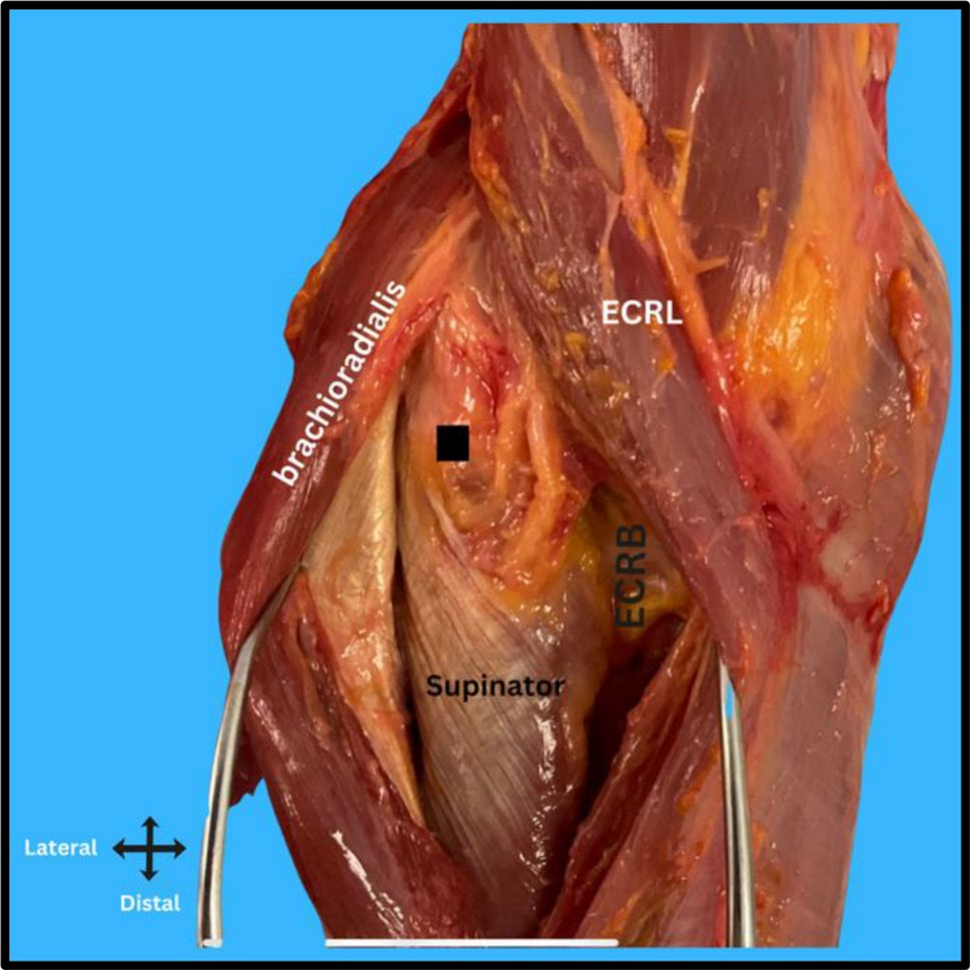
Surgical approach for posterior interosseous nerve (PIN) dissection. The interval between the extensor carpi radialis longus (ECRL) and brachioradialis (BR) is utilized to expose the radial nerve (RN) and its branches. The posterior interosseous nerve is identified as entering the supinator muscle at the arcade of Froshe. A black square highlights the location of the PIN within the dissection field

Data collection and analysis were carried out systematically. Measurements were obtained using a digital caliper (Hoechstmass, Sulzbach, Germany). Two surgeons independently recorded the measurements and resolved discrepancies by consensus. For the dissection and identification of the posterior interosseous nerve (PIN), with the elbow fully extended and the forearm in full supination, a long anterior 20 cm incision was made from the distal arm to the mid forearm, centered over the radial tuberosity. Then the surgical approach followed the interval between the brachioradialis and brachialis proximally, and between the brachioradialis and extensor carpi radialis longus distally (Fig. 2). This interval provided clear visualization of the radial nerve and PIN’s entire course, from its bifurcation from the radial nerve proximally to its entry into the supinator distally. Care was taken to preserve the natural anatomical course of the nerve throughout dissection.

Once the PIN was fully exposed, several measurements were obtained using a digital caliper, including the following: distance from the lateral humeral epicondyle to the radial nerve bifurcation, as well as from the bifurcation point to the radial tunnel. With the elbow in extension and supination, the following parameters were recorded:

- The horizontal distance from the radial nerve to the lateral epicondyle.
- The vertical distance from the PIN bifurcation to the radiocapitellar joint (RCJ).
- The entry point of the PIN into the supinator relative to the radial tuberosity (RT) is classified as proximal, distal, or at the same level.

Additional measurements were performed in both pronation and supination, including:

- The distance from the PIN’s entry into the supinator tunnel relative to the radial tuberosity.
- The vertical distance from the RCJ to the PIN’s entry into the supinator tunnel in pronation.

To evaluate surgical safety, we also examined:

- The potential risk of PIN injury when elevating the supinator from its radial attachment from the ulnar to radial direction.
- The safe positioning of two retractors relative to the proximal and distal parts of the radial tuberosity.

### Anatomical orientation, positioning, and terminology

Forearms were positioned in full supination unless otherwise stated. When describing locations around the radial tuberosity, the terms radial and ulnar are used in preference to lateral/medial. The proximal border of the radial tuberosity refers to the edge closest to the radiocapitellar joint (RCJ), while distal denotes the edge of the tuberosity oriented toward the radial shaft.

Measurements were taken at end-range supination and end-range pronation (defined as the maximal rotation achievable without soft-tissue strain on the setup).

Distances from the radiocapitellar joint (RCJ) were measured from the midpoint of the radiocapitellar articulation. The point of PIN entry was defined at the Arcade of Frohse, where the nerve passes between the superficial and deep heads of the supinator.

Descriptive statistics (mean, standard deviation, range, and 95% confidence intervals) were calculated for the measurements. A paired t-test was used to compare measurements obtained in pronation and supination, with statistical significance defined as *p* < 0.05. Statistical analyses were performed using Microsoft Excel (Version 2021).

## Results

**Fig. 3:**
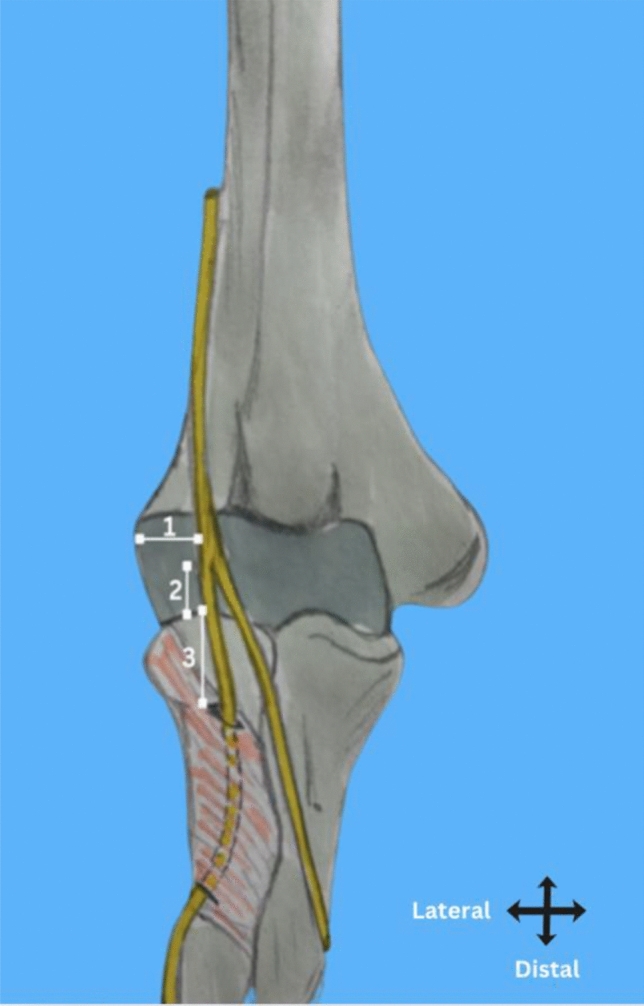
An illustration showing key landmarks of the posterior interosseous nerve (PIN) and its surrounding structures. Number 1 shows the distance between the lateral epicondyle and the radial nerve. Number 2 shows the distance from the bifurcation of the radial nerve to the radiocapitellar joint (RCJ). Number 3 shows the distance from the Arcade of Frohse to the RCJ

**Fig. 4:**
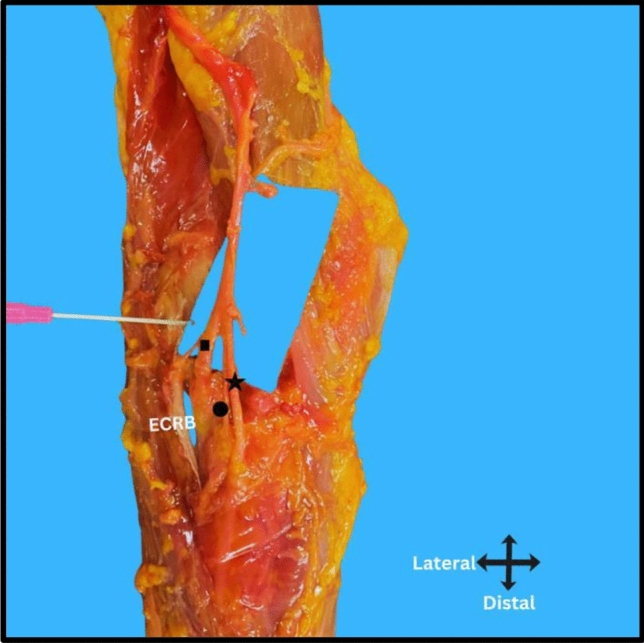
Cadaveric dissection illustrating the relationship between the radiocapitellar joint and the bifurcation of the radial nerve. The pinkish needle is placed at the level of the radiocapitellar joint. The radial nerve divides into the superficial radial sensory nerve (marked with a star) and the posterior interosseous nerve (PIN, marked with a square). The branch to the extensor carpi radialis brevis (ECRB) is indicated with a circle

**Fig. 5:**
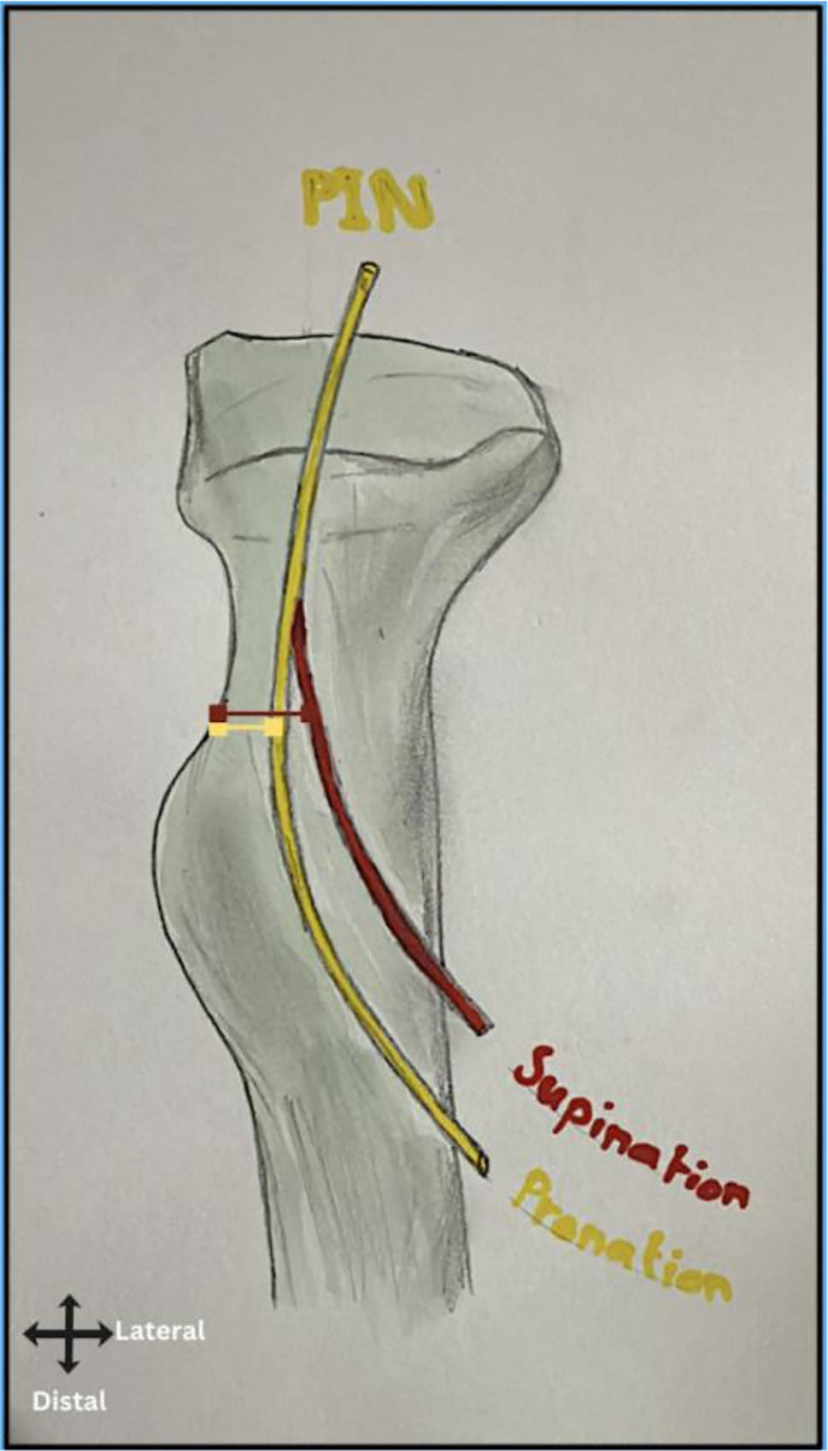
An illustration showing the distance from the radial border of the radial tuberosity to the posterior interosseous nerve (PIN). In pronation (highlighted in yellow), the mean distance was 13 mm, while in supination (highlighted in red), the distance increased to a mean of 19 mm

The radial nerve was positioned at a mean distance of 24 ± 6 mm (SD) medial to the lateral epicondyle, Range: 15–39 mm, 95% Confidence Interval: 22–26 mm (Fig. 3). The bifurcation of the radial nerve into the posterior interosseous nerve (PIN) and the superficial radial nerve (SRN) occurred approximately 5 ± 3 mm (SD) proximal to the radiocapitellar joint ( 95% CI 2–8 mm) (Fig. 3, 4). Following its bifurcation, the posterior interosseous nerve (PIN) entered the Arcade of Frohse at a mean distance of 22 ± 1 mm (SD) distal to the RCJ (range 17–28 mm, 95% CI 21–23 mm) (Fig. 3). In all specimens, the Arcade of Frohse was consistently positioned proximal to the radial tuberosity (Fig. 3). The PIN entered the supinator muscle at a mean distance of 13 ± 2 mm from the lateral border of the radial tuberosity in pronation (range 4–25 mm, 95% CI 12–15 mm), and 19 ± 1.7 (SD) mm in supination (range 10–30 mm, 95% CI 17 –20 mm) (Fig. 5, Table 1). The lateral border of the radial tuberosity refers to the most lateral aspect of the tuberosity as seen in each forearm position, which varies slightly between pronation and supination. Paired t-test, *p* < 0.001; This shows a statistically significant difference between pronation and supination, with the nerve being farther from the radial tuberosity in supination.

**Table 1:** Anatomical measurements of the posterior interosseous nerve (PIN) and clinical relevance. (All distances are reported in millimeters [mm]

Anatomical parameter	Mean ± SD	Range	Surgical implication
Distance from radial nerve to LE	24.0 ± 6.0	15–39	Reference point for locating radial nerve bifurcation
Distance from radial nerve bifurcation to RCJ	5.0 ± 3.0	0–30	Helps identify location of bifurcation during exposure
Distance from RCJ to PIN entry (Arcade of Frohse)	22.0 ± 1.0	17–28	Confirms consistent position of Arcade Proximal to RT
Distance from Arcade of Frohse to radial tuberosity (pronation)	13.0 ± 2.0	4–25	High-risk zone–Supination is recommended
Distance from Arcade of Frohse to radial tuberosity (supination)	19.0 ± 2.0	10–30	Safer zone–Supination is recommended
Muscle coverage distal to Arcade of Frohse	Present in 100%	–	Suggests protective muscular envelope for PIN
Safe retractor placement–proximal tuberosity	–	–	Retractor recommended to be placed medially
Safe retractor placement–distal tuberosity	–	–	Lateral retractor placement acceptable in supination

**Fig. 6:**
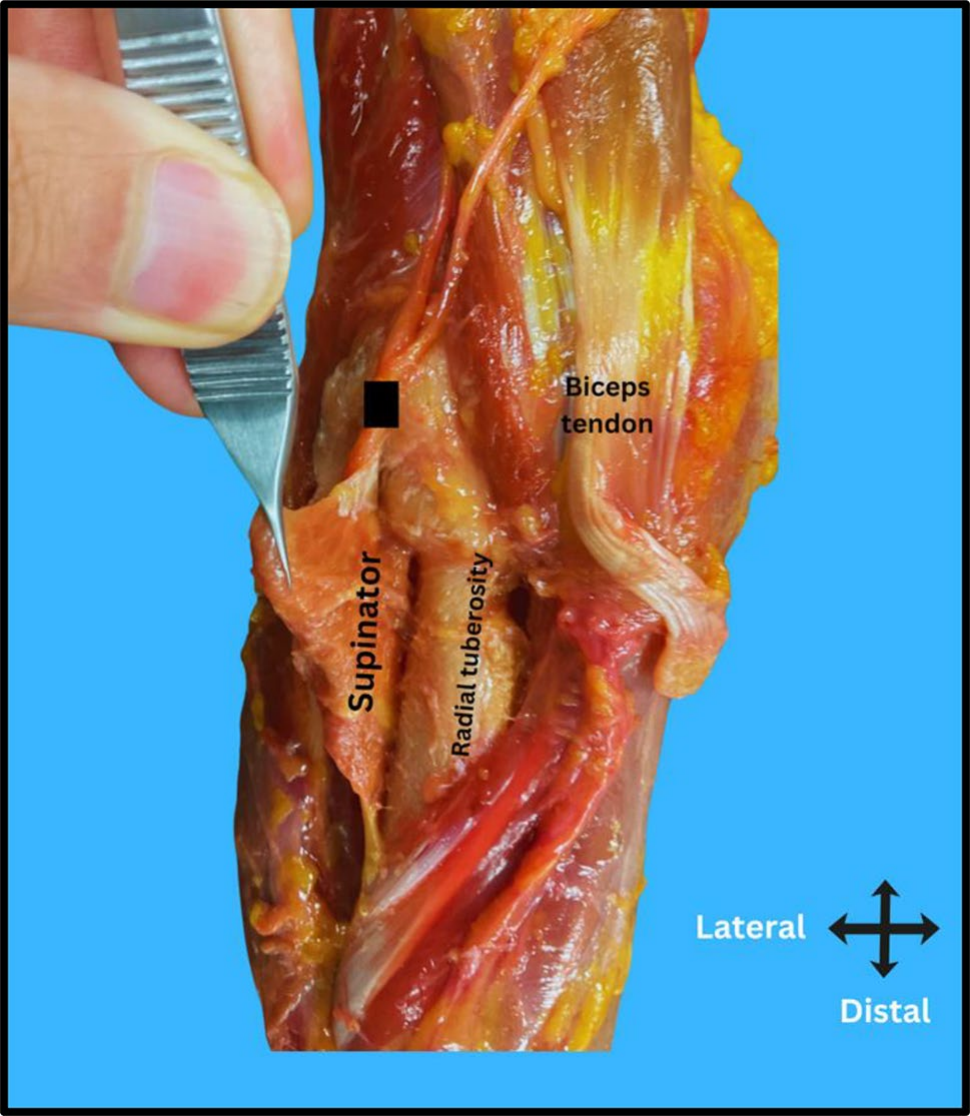
Cadaveric dissection demonstrates that the posterior interosseous nerve (PIN) is surrounded by muscle layers between the superficial and deep heads of the supinator muscle. When the supinator was elevated from ulnar to radial direction, the PIN remained protected within the supinator muscle between the superficial and deep heads of the muscle

In all specimens, the PIN distal to the Arcade of Frohse was enclosed between the superficial and deep heads of the supinator muscle (Fig. 6).

**Fig. 7:**
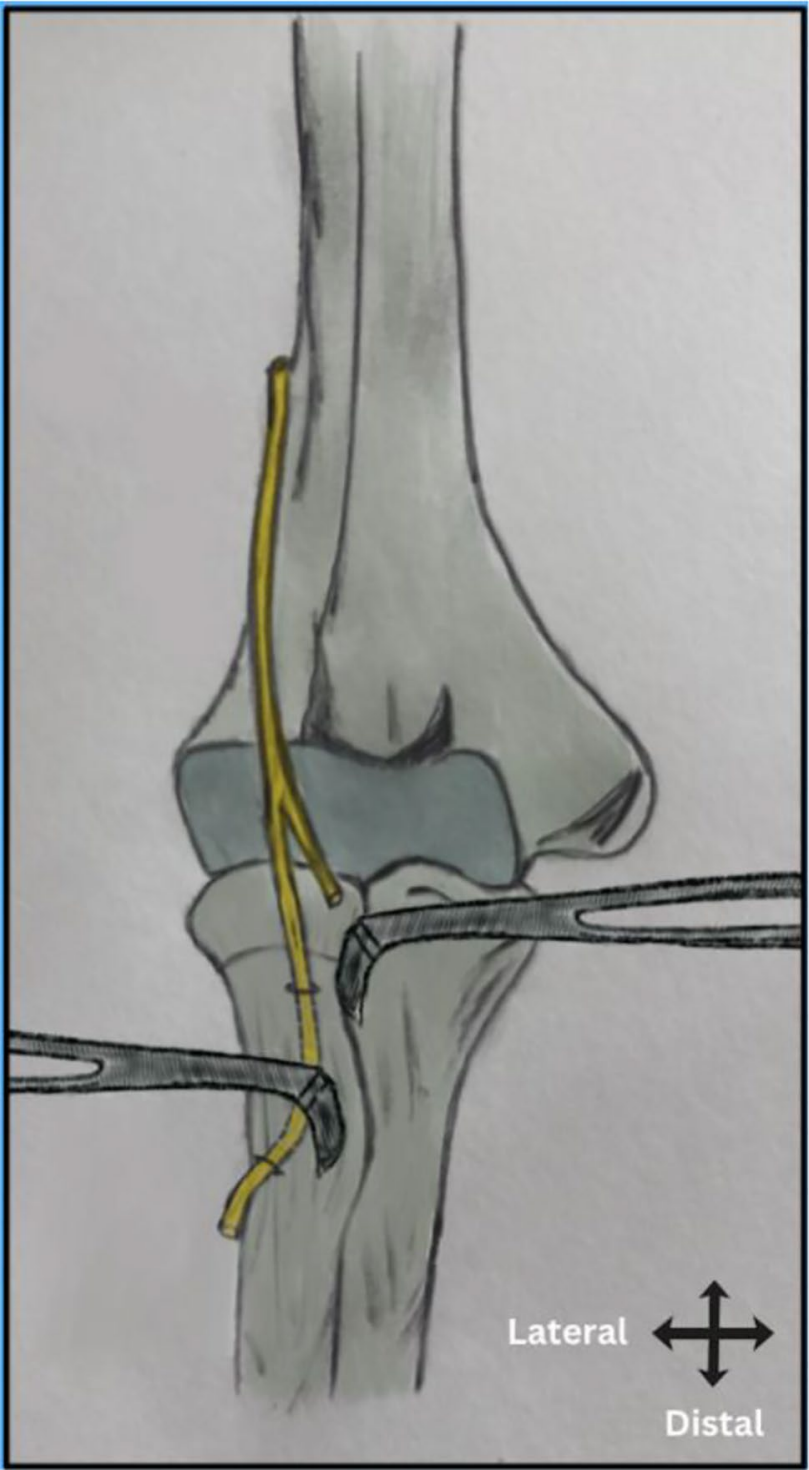
Schematic illustration demonstrating the recommended zones and directions for retractor placement relative to the radial tuberosity. At the proximal border of the tuberosity, the retractor is recommended to be positioned ulnarly to minimize the risk of injury to the PIN, which lies closest to the tuberosity in this region. At the distal border, retractor placement at the radial side is considered safe, as the PIN has already entered the supinator and is positioned farther away from the tuberosity

Based on these anatomical findings, optimal retractor positioning is suggested as follows (Fig. 7):

- Proximally, the retractor may be placed ulnar to the radial tuberosity to minimize risk of nerve injury.
- Distally, retractor placement at the radial border of the radial tuberosity appears safe, with no direct risk to the PIN.

## Discussion

Iatrogenic posterior interosseous nerve (PIN) injury remains a major concern during distal biceps tendon repair, especially with the single-incision anterior approach [3, 14, 20]. Understanding the precise anatomical relationship between the PIN and key bony landmarks, particularly the radial tuberosity, is crucial to minimizing this risk. This cadaveric study provides a detailed assessment of the PIN’s course in relation to the radial tuberosity and other anatomical landmarks, offering new insights into safe surgical retractor placement. The PIN lies closest to the proximal portion of the radial tuberosity and shifts posteriorly as it courses distally. Our findings demonstrate that while the PIN consistently remains surrounded by muscle distal to the Arcade of Frohse, its proximity to the radial tuberosity varies with forearm positioning. Notably, the nerve is positioned closest to the radial tuberosity at its proximal portion and gradually shifts posteriorly as it extends toward the distal part of the tuberosity. These findings support placing retractors ulnar at the proximal tuberosity, while radial placement distally appears safe when the forearm is supinated.

Our study examined the relationship between the PIN and the lateral epicondyle as the nerve traverses this region. We found that the PIN was located an average of 24 ± 5.5 mm medial to the lateral epicondyle. To our knowledge, no previous study has specifically measured this distance for direct comparison. Given that the lateral epicondyle is a readily palpable anatomical landmark, this measurement can serve as a useful reference for approximating the PIN's location at its proximal segment just before it crosses the elbow.

The radial nerve was observed to bifurcate into the posterior interosseous nerve (PIN) and the superficial radial nerve (SRN) at an average distance of approximately 5 ± 3.2 mm proximal to the radiocapitellar joint (RCJ). This finding aligns with previous anatomical studies. For instance, Prasartritha et al. reported a bifurcation level at 13 mm proximal to the RCJ in their cadaveric study [21], while Cox et al. reported a bifurcation distance of 10 mm proximal to the RCJ [7]. These slight variations in bifurcation distance likely result from anatomical differences among specimens and variations in measurement techniques. However, both our findings and those of previous studies consistently demonstrate that the mean distance of bifurcation occurs proximal to the RCJ.

Following its bifurcation, the posterior interosseous nerve (PIN) entered the Arcade of Frohse at an average distance of 22 ± 1.1 mm (SD) distal to the radiocapitellar joint (RCJ). In all specimens, the Arcade of Frohse was consistently positioned proximal to the radial tuberosity. This measurement aligns closely with the findings of Prasartritha et al., who reported a distance of 23 mm from the RCJ to the point where the PIN enters the supinator [21]. Additionally, our results are consistent with those of Schwarz et al., who examined contralateral extremities and documented distances of 25.2 ± 5.9 mm on the right and 24.7 ± 5.6 mm on the left [22].

The PIN’s entry into the supinator muscle (Arcade of Frohse) was located at an average distance of 13 ± 1.6 mm (SD) radial to the radial border of the radial tuberosity in pronation and 19 ± 1.7 mm (SD) in supination. These results suggest that forearm supination may increase the distance between the PIN and the tuberosity, offering a protective effect.

Our results are consistent with the cadaveric study conducted by Schwarz et al., which measured the distance between the distal biceps tendon and the Arcade of Frohse. Their findings showed that in pronation, the distance was 14.1 ± 3.4 mm on the right extremity and 13.5 ± 3.2 mm on the left, whereas in supination, the distance increased to 20.5 ± 3.6 mm on the right and 19.8 ± 3.5 mm on the left [22]. These similar measurements reinforce the idea that supination serves as a protective maneuver, moving the PIN further away from the surgical field and thereby reducing the likelihood of iatrogenic nerve injury.

Whether retractor insertion or drilling is the primary cause of postoperative PIN palsy is still unknown. Drill trajectory during cortical button fixation has a considerable impact on the risk of PIN injury, as shown by Lo et al. [18]. In contrast to drilling instructions, our study concentrated exclusively on retractor safety.

In every cadaveric specimen, distal to the Arcade of Frohse, the PIN was consistently positioned between the superficial and deep heads of the supinator, without exception, the PIN distal to the Arcade of Frohse was consistently between the superficial and deep heads of the supinator muscle, preventing direct contact between the nerve and the periosteal bone at the level of the radial tuberosity.

A crucial surgical consideration is that in every dissection, when the supinator was elevated off from ulnar to radial direction, the PIN remained protected within the superficial and deep head of the supinator muscle. The PIN remained enclosed within muscle layers without direct contact with the radius (Fig. 6). This observation clarifies the anatomical relationship between the PIN and the supinator muscle. This anatomical arrangement allowed the nerve to move within the muscle layers without sustaining injury. To the best of our knowledge, this specific anatomical finding has not been previously described in the literature. This insight provides information regarding the relationship between the PIN and the supinator muscle around the radial tuberosity, emphasizing the protective role of the muscle.

When the PIN is proximal to the radial tunnel (RT), it has not yet entered the supinator muscle and remains unprotected. At this level, the average distance between the radial border of the RT and the PIN is 13 mm, whereas distally, at the distal part of the radial tuberosity, this distance increases to 19 mm. Therefore, proximally, retracting the PT medially while avoiding lateral retraction may help prevent nerve injury.

In contrast, at the distal part of RT, the PIN has already entered the supinator and shifts posteriorly, away from the radial border of the radial tuberosity (mean distance of 19 mm), providing greater protection. At this level, a radial retractor can be placed more safely.

Additionally, our findings indicate that forearm supination increases the distance between the PIN and the RT (Fig. 5). This suggests that maintaining forearm supination during surgery may further lower the risk of PIN injury by increasing the separation between the nerve and a radially positioned retractor. These findings may help guide retractor placement strategies to reduce the risk of iatrogenic nerve injury.

Our cadaveric findings demonstrating increased safety margins in supination are supported by the MRI study of Luthringer et al. [19], which showed that the PIN lies significantly farther from the bicipital tuberosity and drilling trajectories in maximal supination compared with pronation. Their imaging-based evidence corroborates our anatomical dissection results, both highlighting the protective role of supination during anterior single-incision distal biceps repair. While Luthringer et al. quantified nerve-tuberosity distances radiographically, our study extends this knowledge by describing anatomical fresh cadaveric dissection and proposing practical retractor positioning. Together, these findings provide convergent evidence that maximal supination is recommended.

Our findings, considered alongside recent evidence of the segmental vascular supply of the radial nerve and its branches and the presence of clinically relevant anatomic variation, underscore the need for meticulous tissue handling and retractor placement during distal biceps repair to minimize iatrogenic nerve injury [8, 16]. With a history of surgery or substantial scarring, preoperative ultrasonography may be used to map the PIN and variant branches to optimize exposure and mitigate nerve-injury risk [1, 16].

There are several limitations to this study. There is a level of heterogeneity intrinsic to cadaveric studies due to differences in height, build, muscle mass, body fat, soft tissue tension, body mass index, and any relevant medical conditions of the donors. In addition, the dissected cadavers were elderly and would have less muscle bulk relative to younger donors. In addition, this study is limited by its cadaveric nature. No dynamic testing or intraoperative simulation was performed, and only static anatomical relationships were evaluated. In addition, no functional or clinical correlation was available. These limitations should be considered when interpreting the clinical implications of our findings and may limit generalizability. However, exclusively fresh cadavers were dissected to preserve the natural anatomy. Caliper-based measurements may not account for 3D topographical variation in depth or contour. Finally, while our findings provide guidance on retractor safety, clinical trials are necessary to confirm whether these anatomical findings translate into improved surgical safety and reduced nerve injury rates.

## Conclusion

This study highlights the importance of strategic retractor placement to minimize the risk of iatrogenic PIN injury during distal biceps tendon repair. The data suggest that radial retractors should be avoided proximally, while lateral placement is safer distally due to greater nerve separation. Additionally, forearm supination increases the separation between the PIN and radial tuberosity, further reducing the risk of nerve compression. These findings may help guide retractor placement strategies to reduce the risk of iatrogenic nerve injury.

## Data Availability

Raw data were generated at the laboratory of the Medical College of Wisconsin. Derived data supporting the findings of this study are available from the corresponding author [Feras Qawasmi]. On request
